# El atractivo de la especialidad de Medicina Familiar y Comunitaria en la elección de plazas MIR 2023

**DOI:** 10.1016/j.aprim.2023.102699

**Published:** 2023-07-08

**Authors:** Yoseba Cánovas Zaldúa, Ermengol Coma, Francesc Fina, Eulàlia Dalmau-Matarrodona

**Affiliations:** a Direcció d’Atenció Primària i a la Comunitat, Institut Català de la Salut (ICS), Barcelona, España; b Equipo de Atención Primaria Passeig de Sant Joan, Institut Català de la Salut (ICS), Barcelona, España; c Sistemes d’Informació dels Serveis d’Atenció Primària (SISAP), Institut Català de la Salut (ICS), Barcelona, España; d Direcció d’Estratègia i Qualitat, Institut Català de la Salut (ICS), Barcelona, España

Entre el 17 de abril y el 11 de mayo del 2023 ha tenido lugar la elección de las 8.550 plazas de residentes de las 46 especialidades en la convocatoria de Formación Sanitaria Especializada 2022-2023^1^.

Medicina Familiar y Comunitaria (MFyC) es la especialidad con más plazas ofertadas con 2455 (28,7% del total), multiplicando por 18 la oferta media del resto de especialidades, que es de 135 plazas.

Muchas de las plazas de MFyC han sido escogidas en los últimos días y 131 (5,3%) han quedado vacantes, lo que ha generado en redes sociales y medios de comunicación una visión negativa hacia la especialidad, con titulares del tipo «MFyC es la única especialidad que “pincha” en el MIR» o «MFyC es la especialidad menos atractiva».

Con el objetivo de ofrecer un análisis alternativo que compare la desigualdad en la oferta entre MFyC y el resto de especialidades se ha elaborado un indicador que mide la «preferencia competitiva» entre ellas. Por cada especialidad diferente de MFyC, se ha obtenido el número de orden de la última persona que la escogió en la ciudad de estudio, que es la última persona que pudo escoger entre esa especialidad y MFyC. Posteriormente, se han seleccionado todas las personas que escogieron aquella especialidad o MFyC con un número de orden inferior, es decir todas las personas que tuvieron posibilidad real de elección entre estas 2 especialidades. Por último, sobre este conjunto de personas, se ha obtenido el porcentaje que escogió MFyC y el que escogió la otra especialidad, como medida de la preferencia competitiva entre ambas.

Así mismo para analizar la preferencia competitiva entre especialidades se escogieron las 5 ciudades españolas más pobladas (más de 600.000 habitantes) y con la mayoría de las especialidades ofrecidas con el objetivo de obtener una muestra homogénea: Madrid, Barcelona, Valencia, Sevilla y Zaragoza.

En la tabla 1 se muestran los resultados del porcentaje de preferencia de MFyC respecto al resto de especialidades. Valores iguales o superiores al 50% (sombreado claro en versión impresa y color verde en versión digital) indican una preferencia competitiva positiva de MFyC respecto a la otra especialidad. Los valores inferiores al 50% (sombreado oscuro en versión impresa y color rojo en versión digital) indican una preferencia competitiva negativa. Las especialidades han sido agrupadas por especialidades médicas, quirúrgicas y laboratorios y servicios centrales.

**Tabla 1:**
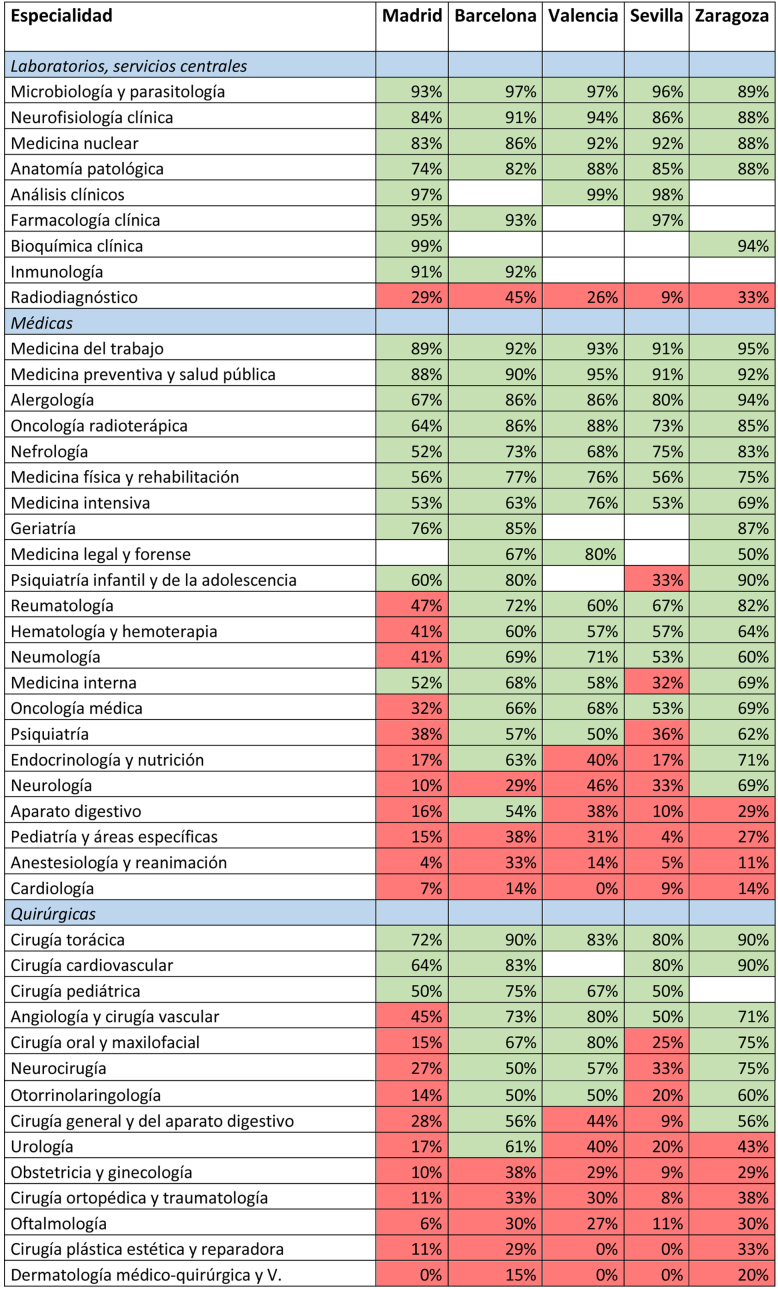
Preferencia competitiva de MFyC respecto al resto de especialidades.

En Barcelona, MFyC presenta una preferencia competitiva positiva respecto al resto de especialidades en 33 de las 44 elegibles (75%). En Zaragoza en 30 de las 42 (71%). En Valencia en 25 de las 40 (62%). En Sevilla en 21 de las 42 (50%). Finalmente, en Madrid, MFyC presenta una preferencia competitiva positiva en 21 de las 45 (46%).

MFyC presenta una preferencia competitiva positiva en la mayoría de las especialidades de laboratorio y servicios centrales. Entre las especialidades quirúrgicas, presenta una preferencia competitiva positiva respecto a cirugía torácica, cirugía cardiovascular y cirugía perdiátrica. Entre las especialidades médicas, MFyC presenta una preferencia competitiva positiva en todas las ciudades analizadas respecto a 10 especialidades (Alergología, Medicina del Trabajo, Medicina Física y Rehabilitación, Medicina Intensiva, Medicina Nuclear, Medicina Preventiva, Nefrología, Neurofisiología, Oncología Radioterápica, Farmacología, Geriatría y Medicina Forense), y respecto a 6 más (Psiquiatría Infantil, Reumatología, Hematología, Neumología, Medicina Interna y Oncología Médica) en 4 de las 5 ciudades analizadas.

Cabe destacar que un buen profesional, y especialmente en el caso de MFyC, se distingue por un conjunto de características que van más allá del acierto obtenido en un examen de preguntas tipo test donde influyen además factores como la Facultad de Medicina donde se ha estudiado^2^ o el esfuerzo requerido por la expectativa de nota necesaria, lo que lleva a preguntarse si el actual modelo de examen y elección de plazas MIR deberían ser replanteados^3^.

Este análisis no quiere perder de vista la necesidad de continuar introduciendo mejoras que hagan realidad una atención primaria accesible, longitudinal y de calidad^4^ en la que tenemos evidencia que la especialidad de MFyC es atractiva entre los candidatos MIR.

## Consideraciones éticas

Los datos para realizar dicho trabajo han sido obtenidos de la sección de Formación Sanitaria Especializada en la página web del Ministerio de Sanidad

## Financiación

El presente trabajo no ha recibido ayudas específicas provenientes de agencias del sector público, sector comercial o entidades sin ánimo de lucro

## Conflicto de intereses

Ninguno.
